# Supplementary figures and images for: Anti-TSH receptor antibodies (TRAb): Comparison of two third generation automated immunoassays broadly used in clinical laboratories and results interpretation
Source: PLoS One. 2022 Jul 25;17(7):e0270890. doi: 10.1371/journal.pone.0270890 (PMC9312426; doi:10.1371/journal.pone.0270890)

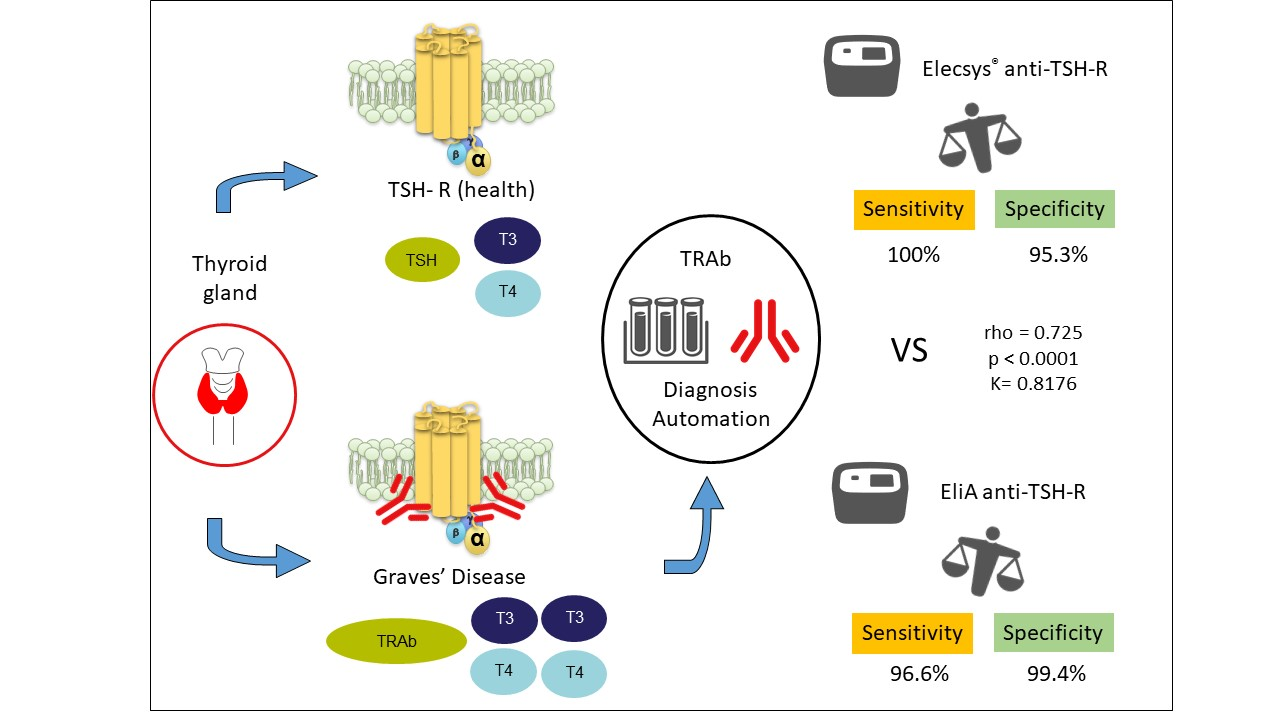

Supplement: S1 Graphical abstract — (TIF) [file pone.0270890.s001.tif]
